# Embryonal Tumors of the Central Nervous System in Children: The Era of Targeted Therapeutics

**DOI:** 10.3390/bioengineering5040078

**Published:** 2018-09-23

**Authors:** David E. Kram, Jacob J. Henderson, Muhammad Baig, Diya Chakraborty, Morgan A. Gardner, Subhasree Biswas, Soumen Khatua

**Affiliations:** 1 Section of Pediatric Hematology-Oncology, Department of Pediatrics, Wake Forest School of Medicine, Winston Salem, NC 27157, USA; 2 Pape Family Pediatric Research Institute, Department of Pediatrics, Oregon Health and Science University, Portland, OR 97239, USA; hendejac@ohsu.edu; 3 Department of Pediatrics, MD Anderson Cancer Center, Houston, TX 77030, USA; MBaig@mdanderson.org (M.B.); DChakraborty@mdanderson.org (D.C.); subhabis@gmail.com (S.B.); SKhatua@mdanderson.org (S.K.); 4 Department of Pediatrics, Wake Forest School of Medicine, Winston Salem, NC 27157, USA; magardne@wakehealth.edu; 5 Humanitas University Medical School, 20090 Milan, Italy

**Keywords:** central nervous system, embryonal tumors, children, medulloblastoma, ATRT, ETMR, molecular biology, targeted therapeutics

## Abstract

Embryonal tumors (ET) of the central nervous system (CNS) in children encompass a wide clinical spectrum of aggressive malignancies. Until recently, the overlapping morphological features of these lesions posed a diagnostic challenge and undermined discovery of optimal treatment strategies. However, with the advances in genomic technology and the outpouring of biological data over the last decade, clear insights into the molecular heterogeneity of these tumors are now well delineated. The major subtypes of ETs of the CNS in children include medulloblastoma, atypical teratoid rhabdoid tumor (ATRT), and embryonal tumors with multilayered rosettes (ETMR), which are now biologically and clinically characterized as different entities. These important developments have paved the way for treatments guided by risk stratification as well as novel targeted therapies in efforts to improve survival and reduce treatment burden.

## 1. Introduction

Over the past decade, a surge genomic and epigenomic data on embryonal tumors of the central nervous system (CNS) in children has dramatically advanced the understanding of tumor biology, paving the way toward improved diagnostic, reclassification, and therapeutic approaches to these formidable malignancies [1]. These new molecular phenotypes have been incorporated in the 2016 World Health Organization (WHO) classification of CNS tumors, creating a major shift in paradigm of the classification of embryonal tumors of the CNS. The 2016 WHO classification integrated genetic information to already-existing histopathological data and has enabled more precise classification and diagnosis of these tumors [2]. This is most evident in the recognition of the molecular subtyping of medulloblastoma (MB), each subtype carrying unique demographics and clinical outcomes. Until recently, all non-medulloblastoma embryonal tumors were encompassed under the umbrella of CNS-primitive neuroectodermal tumors (PNET). Revelations from several molecular-profiling and methylation assay studies now show that a range of distinctly biologically heterogeneous tumors exist that are now known as non-medulloblastoma embryonal tumors of the CNS. The main non-medulloblastoma embryonal tumors include atypical teratoid/rhabdoid tumors (ATRT) and embryonal tumors with multilayered rosettes (ETMR). Other CNS embryonal tumors include the following morphological subtypes: medulloepithelioma, CNS neuroblastoma, CNS ganglioneuroblastoma, and ependymoblastoma [3]. All variants of embryonal tumors are now well-defined entities with varying molecular biology and prognosis. The critical issue is proper diagnosis of these entities, which would allow tailored therapy, including intensifying treatment for aggressive variants and de-escalating therapy for those tumors with better prognoses, in order to achieve long-term cures and minimizing treatment-related toxicity. The following section will discuss the major subtypes of pediatric embryonal tumors, which include MB, ATRT, and ETMR, their molecular biology, and the insights it provides in developing targeted therapies.

## 2. Medulloblastoma

### 2.1. Introduction

MB is the most common pediatric CNS malignancy, accounting for approximately 20% of all pediatric CNS tumors [4,5]. Despite the conventional aggressive multimodal therapeutic approach involving surgery, radiotherapy, and chemotherapy, approximately one-third of patients with MB die from their disease [6]. Survivors often experience long-term sequelae, including neurocognitive deficits, endocrinopathies, and secondary malignancies [7,8,9,10]. Integrative genomic and methylomic studies over the past 15 years have shown that MB is not a single entity, but rather a heterogeneous group of diseases with unique clinical, molecular, and prognostic characteristics [11,12,13,14]. The internationally accepted subgroups of MB have been termed wingless (WNT), sonic hedgehog (SHH), Group 3, and Group 4, which have been adopted in the 2016 WHO classification of central nervous system tumors [2,15]. These efforts to characterize MB have revolutionized our understanding of its pathogenesis and response to treatment, and will hopefully lead to improvements in survival and survivors’ quality of life.

### 2.2. Molecular Subgroups

**Figure 1:**
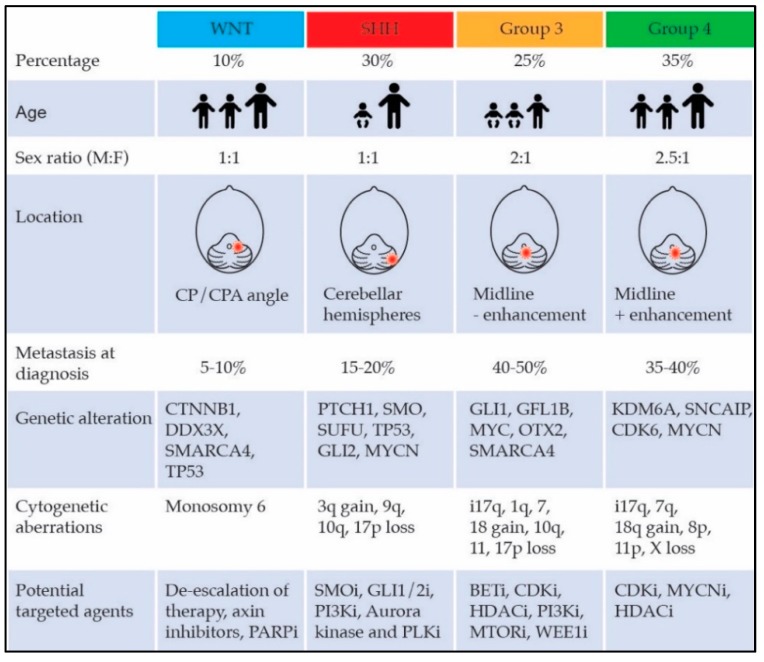
Molecular subgroups of medulloblastoma with unique clinical, genetic, and prognostic characteristics. CP/CPA, cerebellar-pontine/cerebellar-pontine angle; SHH, sonic hedgehog; WNT, wingless and int.

MB subgroups exhibit distinct biological characteristics, and recent application of advanced algorithms for integrative genomic analysis has highlighted heterogeneity even within these subgroups, which may help inform patient stratification in future trials (Figure 1) [16].

WNT: WNT MBs are characterized by activation of the WNT signaling pathway, commonly caused by somatic mutations in the CTNNB1 gene, results in stabilization of the beta-catenin protein [17,18]. WNT MBs are the rarest subgroup, almost never present with metastatic disease, and carry the most favorable prognosis of all the subgroups [12,15,19].

SHH: SHH MBs are characterized by hyperactivation of the SHH signaling pathway, often due to germline or somatic mutations or amplifications in components of the SHH pathway. SHH MBs represent approximately 30% of all MBs and are associated with a markedly variable prognosis [20,21,22,23,24,25]. Of note, upwards of a quarter of patients with SHH tumors have an underlying germline mutation including TP53 or BRCA, which ought to prompt clinicians to consider genetic counseling consultation in all patients diagnosed with SHH MB [26].

Group 3: A unifying pathway has not been identified in Group 3 MBs, as these tumors contain few recurrent somatic and germline mutations aside from amplification of the proto-oncogene MYC and isochromosome 17q [14,22,23,27]. Group 3 MBs account for about 25% of all MBs, affect almost exclusively infants and young children, and have the worst prognosis.

Group 4: The underlying biological abnormalities characteristic of this subgroup are the least understood. Isochromosome 17q is commonly found, but unlike in Group 3 MB, this feature is not associated with poor prognosis [28]. Similar to SHH and Group 3 MBs, there is intertumoral heterogeneity in Group 4 MB [16]. Group 4 is the most common subtype and, despite their common presentation with metastatic disease, overall prognosis of this subgroup is intermediate.

### 2.3. Standard Therapy

**Figure 2:**
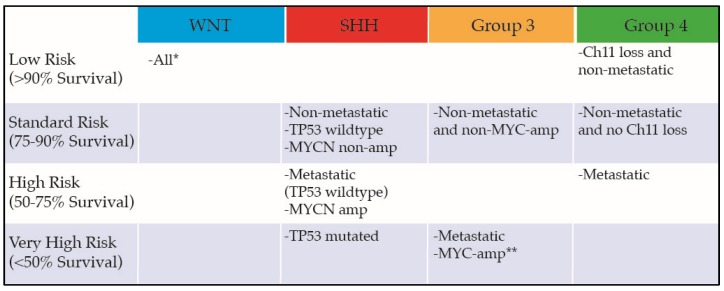
Current risk-stratification schema. * Metastatic WNT tumors are rare and have an unknown natural history. ** Nonmetastatic MYC-amplified tumors are likely very high risk and often recur in a metastatic pattern, but their upfront prognosis is not clear. Ch11, chromosome 11.

Risk stratification: Historically, MB was classified as “average risk” or “high risk” based on age, presence of metastasis at diagnosis, and extent of residual tumor after resection. However, with the recognition of MB subgroups and their respective and independent impact on prognosis, a new risk-stratification schema has been proposed (Figure 2) [29].

Standard treatments: Current therapy for MB consists of maximal tumor resection, craniospinal irradiation (age permitting), and multiagent chemotherapy. The historical goal was a gross total resection, but when controlling for molecular subgroup, the extent of resection may be less important [29]. Radiation is an effective component of MB treatment, though it comes with acute and long-term complications [10,30]. Lower doses may be utilized for certain patients, and conformal, intensity-modulated approaches, as well as electron- and proton-based therapies, can mitigate some toxicities [31,32,33,34,35]. Chemotherapy typically includes the cyclophosphamide/vincristine/cisplatin combination, while radiation-sparing approaches for infants employ combinations of high-dose chemotherapies, often with autologous stem-cell rescue [31,36].

### 2.4. Molecularly Targeted Therapy

WNT: WNT/β-catenin pathway overexpression is the hallmark of WNT MB, which may lend these tumors to be susceptible to axin or PARP inhibitors. Beyond targeted approaches, the favorable outcomes in patients with WNT MB have encouraged efforts aimed at de-escalation of first-line therapy. The Children’s Oncology Group (COG) study ACNS1422, St. Jude’s (SJ) study SJMB12, and the International Society of Paediatric Oncology’s PNET 5 study all reduce the doses of craniospinal irradiation, while the Johns Hopkins J1403 trial was designed to eliminate it altogether [37,38,39]. 

SHH: Efforts to target the SHH pathway have focused on inhibiting the transmembrane receptor smoothened (SMO). Several SMO inhibitors have been introduced into clinical studies, including sonidegib and vismodegib, which have shown safety and improved progression-free survival when used as monotherapy, but both almost universally succumb to development of resistance [40,41,42,43,44,45]. Vismodegib is now being studied in combination with conventional therapy in an ongoing phase 2 study (SJMB12) [37]. Another compelling target is the downstream transcription factor, glioma-associated oncogene (GLI) [46].

Groups 3 and 4: The significant heterogeneity of the biological drivers of these MB subtypes has impaired the development of a clinically applicable targeted approach. However, the understanding that MYC-driven MBs rely on the kinase WEE1 to maintain cell-cycling viability has drawn interest, and an ongoing phase 2 trial (COG ADVL1312) is studying a WEE1 inhibitor in combination with irinotecan [47].

Nonspecific Subgroup Molecular Targeting and immunotherapy: Several other drivers of MB have been identified, though they do not yet fall into any single MB subgroup. The Notch signaling pathway has been implicated in MB tumorigenesis and may be targetable with γ-secretase inhibitors [48]. The PI3K/AKT/mTOR pathway is instrumental in multiple metabolic and survival pathways, and disturbances to this pathway have been implicated in MB [49,50]. Currently, the Pediatric MATCH trial is testing a dual PI3K/mTOR inhibitor in MBs with PI3K/TSC/mTOR mutations [51]. PI3K, histone deacetylase, and BET-bromodomain inhibitors, especially in MYC-amplified MB, show early preclinical promise and are being studied in an early-phase study (PBTC-026) [52,53,54]. Cyclin-dependent kinase 6 (CDK6) is commonly mutated in SHH, Group 3, and Group 4 MB [55]. Inhibitors of CDK4/6 are being studied in ongoing early-phase trials (PBTC-042 and SJDAWN) [56]. Activation of the RAS/MEK/ERK pathway has been observed in MBs, and targeting this pathway is being studied utilizing EGFR, VEGF-A, and MEK inhibitors [57,58,59,60,61].

Like many other pediatric tumors, the potential to harness the immune system’s innate antitumor effect to treat MB is being increasingly appreciated. Early studies have shown that most MB tumors have only nominal immune-cell infiltration and tumor cells have absent or deficient antigen-presenting machinery [62,63]. Despite the apparent lack of tumor surveillance, there is growing evidence that checkpoint inhibition, immunization, or viral therapy may be of benefit [64,65,66].

### 2.5. Conclusion

Over the past 15 years, our understanding of MB has greatly increased, enabling the classification of subgroups. Subgroup-based risk stratification offers the potential to improve overall survival while reducing treatment burden in lower risk patients. The continuous discovery of biological diversity within MB subgroups, however, makes risk assignment and selection of appropriate patients for novel therapies more challenging [20]. Furthermore, the genomic and transcriptomic profiling of a tumor is likely spatially variable and is dynamic with treatment. By profiling MBs as bulk samples, there is risk of masking potential intratumoral heterogeneity, missing data of MB stem-cell programs, and concealing information about nonmalignant cells of the tumor microenvironment. MB is a heterogeneous disease, and treating all patients with standard therapy is no longer acceptable. However, while the era of targeted therapies based on genetic perturbations has great promise, future clinical trials will require novel approaches to best pair potent therapies to susceptible tumors.

## 3. Atypical Teratoid Rhabdoid Tumors (ATRT)

### 3.1. Introduction

ATRTs represent a variant of embryonal tumors of the CNS affecting younger, predominantly male children. They account for 1–2% of CNS tumors in children, with a peak incidence of children aged less than 3 years. Two-thirds of these neoplasms occur in the cerebellum, commonly at the cerebellar-pontine angle (CPA), with a potential of multifocal or disseminated disease in 20% of patients at the time of diagnosis [67]. Prognosis remains poor, though recent revelations of biological data and improved understanding of the signaling pathways now offer some therapeutic optimism [68]. Recent treatment strategies have focused on using radiation and chemotherapy along with targeted therapeutics, which could improve overall prognosis [69,70].

### 3.2. Clinical Features and Diagnosis 

Clinical presentation of ATRT largely depends on the location of the tumor and metastatic status. These tumors do not have extraordinary neuroimaging characteristics; their appearances are quite similar to medulloblastoma. Their appearance is often heterogeneous with high cellularity, containing a distinct band of wavelike enhancement; they may have intratumoral hemorrhage and peripherally located cysts; they may also show patterns of restricted diffusion [71]. Recent findings suggest that MRI features may vary across different molecular subgroups of ATRT [72,73]. ATRT morphology is diverse, ranging from epithelial to mesenchymal to neuroepithelial, and sometimes containing all three. Classic rhabdoid cells are less prevalent in these tumors, and instead more commonly exhibit small-blue-round-cell tumor morphology, similar to medulloblastoma. Prior to the discovery of the SMARCB1 mutation as a specific molecular marker for ATRT, they were diagnosed under the same umbrella as PNET or medulloblastoma [74]. Currently accepted diagnostic criteria include biallelic loss and/or negative immunohistochemistry staining of SMARCB1 or SMARCA4 and their respective gene products hSNF/INI1/BAF47 and BRG1 [75,76]. Importantly, 20–35% of patients with ATRT are found to carry biallelic germline alterations of SMARCB1 or SMARCA4. These patients have rhabdoid tumor predisposition syndrome, with a propensity to develop intra- and extracranial aggressive rhabdoid tumors at young age [77,78]. Thus, all patients with ATRT should undergo genetic counseling and testing for the presence of a germline mutation.

### 3.3. Molecular Era of ATRT

The sole recurrent genetic alterations in these tumors are biallelic mutations of SMARCB1 (INI1, SNF5, BAF47) or, rarely, SMARCA4, both of which are members of the SWItch/Sucrose Nonfermentable chromatin-remodeling complex [79]. Approximately 20% of the 22q11.2 deletions include SMARCB1, of which 25% of the patients have partial deletion or duplication. The remaining chromosomal aberrations include single-base point mutations, frame shifts, or insertions [80].

**Figure 3:**
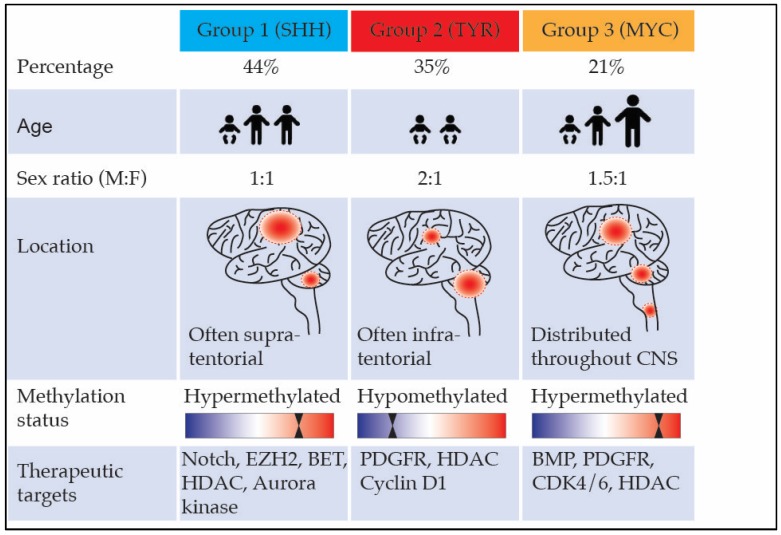
Molecular subgroups of atypical teratoid rhabdoid tumors (ATRT) and their clinical and epigenetic features, as well as therapeutic targets.

High-resolution molecular studies have uncovered marked clinical and molecular heterogeneity in the relatively bland genome of ATRT. Varying molecular subgroups in ATRT were first identified by Birks et al., who showed high expression of bone morphologic protein (BMP) in a subgroup associated with shorter survival [81]. A subsequent larger study, using integrated analysis of clinical and transcriptional data, demonstrated two major subtypes with different clinical outcomes: the supratentorial tumors, which were characterized by neuronal differentiation and ASCL1 protein expression, correlated with improved survival outcome, while infratentorial tumors enriched with BMP signatures carried worse prognosis [82]. A recent study utilizing genetic, epigenetic, and transcriptional characterization subdivided ATRT into three methylation subgroups with varying demographics and molecular profiles. These are ATRT-SHH, ATRT-TYR, ATRT-MYC [79]. Figure 3 shows the molecular subtypes including demographics, SMARCB1 profile, epigenetic features, and therapeutic targets of interest. The generation of the first transgenic mouse model harboring temporal deletion/inactivation of SMARCB1 facilitated further understanding of the oncogenic events leading to ATRT formation. This study revealed that epigenetic mechanisms associated with hSNF5 loss drives these tumors and provided insights into the different targeted cells of origin that likely contribute to the heterogeneous nature of ATRT [83]. 

### 3.4. Current Treatment

Given the rarity of the disease and the diversity of treatment regimens historically employed to treat ATRT, no standard therapeutic approach exists. There seems to be improved survival for those patients with de novo ATRT treated with Intergroup Rhabdomyosarcoma Study (IRS) protocols and high-dose alkylating agents [84]. A phase II study by the Dana Farber Cancer Institute treated 20 patients with ATRT using a modified IRS III approach. Patients on this study had good outcomes, with an OS of 70% over two years, which was improved in comparison to other historical data [85]. A retrospective study by St. Jude Cancer Research Hospital included 31 children older than three years with ATRT who received craniospinal irradiation, high-dose alkylator-based chemotherapy, and showed that they fared better, with a two-year OS of 89 ± 11% [86]. The role of high-dose chemotherapy (HDC) followed by stem-cell rescue in studies by the Head Start and the Canadian Brain Tumor Consortia showed improved outcomes with intensive chemotherapy regimens, though toxicity was not inconsequential [87,88]. ATRT registry data suggest that the extent of surgical resection correlates uniformly with improved survival [89]. The role of radiotherapy still remains unclear and in general has been deferred or dose-reduced in younger children with ATRT. However, recent studies have shown a trend towards increased survival time with the addition of radiotherapy, leading to its incorporation in various clinical trials [90,91]. The current COG ACNS0333 trial (NCI: NCT00653068) incorporates a combination of chemotherapy and radiation therapy, along with autologous stem-cell rescue in treating young patients with ATRT.

### 3.5. Newer Therapeutic Insights

It is now evident that ATRT is an epigenetically driven disease, and thus targeting oncogenic drivers in epigenetic machinery is now the key focus of developmental therapeutics against these malignancies. The finding that ATRT may exhibit increased expression of EZH2, and subsequent hypermethylation has led to preclinical testing of EZH2 inhibitors EPZ-6438, 3-deazaneplanocin, and tazemetostat, alone or in combination with other chemotherapies; the data are encouraging, showing some tumor responsiveness to this approach [92,93]. A phase I trial using tazemetostat monotherapy against hypertrimethylated tumors, including one with an INI1-deleted malignant rhabdoid tumor, showed promising results: the one ATRT-like patient achieved a partial response [94]. Upregulation of the Bromo/BET domain may also contribute to ATRT oncogenesis, and Bromo/BET inhibitors have shown some promise in preclinical models [95,96]. Histone deacetylase inhibitors, which are also epigenetic modifiers, have been successfully used to inhibit ATRT growth and increased sensitivity to radiation [97]. Aurora kinase A (AURKA) is highly expressed in ATRT because of the loss of the INI1 tumor-suppressor gene. Alisertib, an AURKA inhibitor, has been explored in ATRT with good outcomes in patients with progressive disease [69]. The association between INI1 loss and increased expression of cyclin D1 in nearly 80% of ATRTs led to testing cyclin D1 as a druggable target [98,99]. CDK4/6 inhibitor palbociclib, when used in combination with radiation in preclinical models, showed delayed growth of ATRT cells [100]. Following this finding, a clinical trial employed another CDK4/6 inhibitor, ribociclib, alone or in combination with conventional chemotherapy in a few patients with ATRT, and it has shown some initial success [101]. The highly heterogeneous nature of these tumors led to the possibility of exploring multitargeted tyrosine kinase inhibitors (TKI). A recent study showed second-generation TKIs nilotinib and dasatinib reducing cellular proliferation of these tumors, through downregulation of PDGFRβ, and more so in the clinically worse subtype 2 [79]. 

### 3.6. Conclusions

ATRT is an aggressive malignancy with poor overall survival in metastatic disease and in younger children. Though multiple therapeutic approaches have been pursued over the last two decades, prognosis has remained grim until recently. It is now suspected that epigenetic alterations may be important druggable targets, and a variety of trials testing this hypothesis are ongoing. Recent identification and description of molecular subclasses of ATRT should guide future risk-stratified and targeted therapeutic approaches with the intent of reducing toxicity and improving overall survival of these formidable tumors.

## 4. Embryonal Tumor with Multilayered Rosettes (ETMR)

### 4.1. Introduction

ETMR was first identified in 2009 as an aggressive embryonal tumor with a unique molecular phenotype occurring in younger children. Histologically, it represents a PNET with ependymoblastic rosettes and neuropil-like areas containing neurocytes and ganglion cells [102]. ETMR can exhibit three distinct histological patterns: embryonal tumors with abundant neuropil and true rosettes (ETANTR), ependymoblastoma (EBL), and medulloepithelioma (MEPL). The uniform amplification of C19MC in these three distinct histologies has led to the grouping of ETANTR, EBL, and MEPL into a single diagnostic entity known as ETMR [103,104].

### 4.2. Clinical Features and Diagnosis

Due to the rarity of ETMR and limited number of publications, robust demographics have not been defined. These tumors have a very aggressive clinical course, with a median survival of 12 months after diagnosis. ETMR usually occurs in children younger than four years, and is more common in girls, unlike the other CNS embryonal tumors, in which boys are equally or more commonly affected. Clinical features are determined by the location and extent of the tumor. Most are supratentorial in location, a few are infratentorial, and they are very rarely encountered in the spinal cord. Increased intracranial pressure, seizures, hemiparesis, cerebellar signs, cranial nerve palsies, and other neurologic deficits have been reported. In general, neuroimaging shows these tumors as large, demarcated, solid masses featuring patchy- or no-contrast enhancement with surrounding edema, often with significant mass effect. A minority of the reported cases have shown cystic components and microcalcifications [105,106]. Nearly 80% are localized at diagnosis, but even these often rapidly progress. The diagnosis of ETMR remains challenging with only histopathological reviews. LIN28A evaluation by immunohistochemistry remains confirmatory. Comparative genomic hybridization array for amplification of the microRNA cluster C19MC at locus 19q13.42 may further secure the diagnosis [107].

### 4.3. Current Treatment Strategies

Current treatment protocols include maximal-safe surgery with subsequent chemotherapy, often including HDC with stem-cell rescue and focal or craniospinal irradiation depending on the age of the patient and extent of the tumor. Conventional chemotherapeutic agents used include cyclophosphamide, methotrexate, vincristine, etoposide, and carboplatin. Due to the small number of ETMR patients reported in the literature, and variable treatment strategies rendered, it is difficult to draw robust conclusions on the benefits of different treatment regimens on clinical outcome. Though initial responses were seen in some cases, the aggressive nature of these tumors resulted in early progression or relapses and poor overall survival. Despite intensive multimodality therapy with aggressive surgery, chemotherapy, and radiation, five-year overall survival has been less than 10% [108]. Extent of resection seems to be a favorable prognostic factor, though, in another series, radiation therapy correlated with improved outcome [109]. The impact of HDC on long-term survival is still not clear due to the limited number of patients treated with this modality. Lack of biographical input of these tumors has undermined development of targeted therapies and improved survival. However, recent availability and studies on cell lines and preclinical models are now uncovering promising targets for these lethal tumors.

### 4.4. Molecular Characteristics and Therapeutic Insights 

The uniformity of C19MC amplification in these tumors led to the restructuring of ETMR classification. Transcriptional signatures of C19MC-altered tumors reveal enrichment for early neural and pluripotency genes, including LIN28/LIN28B. This suggests the primitive nature of these malignancies and may explain their aggressive phenotype [110]. Global molecular studies identified LIN28A immunoexpression as a highly specific marker for ETMR, with a hallmark genomic amplification of the C19MC oncogenic miRNA cluster. Nearly 25% of ETMR tumors with the molecular features of C19MC alterations are devoid of classical rosette structures, indicative of intratumoral heterogeneity. Global methylation assay and exome-sequencing studies have failed to identify other recurrent alterations, reaffirming C19MC as the main oncogenic driver of these formidable tumors. Though highly suggestive of ETMR, enrichment of LIN28/LIN28B is not specific for ETMR, as this is also seen in ATRT and high-grade gliomas [111].

Lack of optimal preclinical ETMR models has historically precluded defining conventional or novel targeted therapies. However, recent establishment of ETMR cell lines has led to in vitro and in vivo preclinical drug screening. The MTOR pathway appears to be markedly overexpressed in these tumors and is sensitive to inhibition by mTOR inhibitors demonstrated in cell lines [104]. RNA sequencing identified recurrent gene fusions of C19MC miRNAs and neural-specific DNMT3B isoforms in these ETMR tumors. These findings suggest DNMT3B as an important downstream effector of the C19MC oncogene and a potential therapeutic target for these formidable tumors [110].

Recent development of patient-derived xenograft models is now also providing avenues to explore drug activity in vivo. A mouse model of ETMR shows parallel activation of SHH and WNT signaling. This confirms that coactivation of these pathways in mouse neural precursors is sufficient to induce ETMR tumors [112]. These neoplasms resemble human counterparts, both histopathologically and by global gene-expression analysis, suggesting this as a feasible mouse model for further biological interrogation. These tumors responded to the SHH inhibitor, arsenic trioxide, proposing downstream inhibition of SHH signaling as a therapeutic option for patients with ETMR. The role of combination chemotherapy in the ETMR xenograft mice model has been recently evaluated. A combination of topotecan or doxorubicin, along with methotrexate and vincristine, demonstrated longer survival of animals in the topotecan arm. This study also highlighted a potential role for epigenetic modifying drugs, such as panobinostat, and targeted drugs, such as the pololike kinase 1 inhibitor, volasertib, in these tumors [113]. These biological revelations provide the initial clues to profile treatment using a targeted therapeutic approach with conventional chemotherapeutic agents.

### 4.5. Conclusions

C19MC-altered tumors, now identified as ETMR, carry a distinct aggressive clinical course in the setting of conventional therapeutic strategies. However, improved understanding of biological/molecular data aided by preclinical studies now provides avenues to render novel targeted therapies. Efforts to identify genomic and epigenetic machinery driving C19MC-associated tumorigenesis should continue, which would help in developing more precise, less toxic, and optimal treatment for these potentially lethal tumors.
